# Supplementary material for: SHADeS: self-supervised monocular depth estimation through non-Lambertian image decomposition
Source: Int J Comput Assist Radiol Surg. 2025 May 13;20(6):1255–63. doi: 10.1007/s11548-025-03371-8 (PMC12167237; doi:10.1007/s11548-025-03371-8)
Supplement: Supplementary file 1 — (pdf 46712 KB) [file 11548_2025_3371_MOESM1_ESM.pdf]

1 Metrics

Specularity Surrounding Metric (SSM) evaluates the similarity between specular and surrounding regions in the estimated depth. First, the mean intensities of the specular pixels ($Mean_{spec}$) and the surrounding pixels ($Mean_{surr}$) are calculated in Equation 1. The relative difference between these means ($diff_k$) is then computed for each specularity k in Equation 2. If this difference is below a set threshold (1%), the specularity is considered similar to its surrounding area, indicated by $N_{sm}[k] = 1$; otherwise, $N_{sm}[k] = 0$ as shown in Equation 3. Finally, the percentage of specularities that meet this similarity condition is given by P_{sm} in Equation 4.

$$Mean_{spec} = \frac{1}{N}\sum_{i=1}^{N} d_{spec}[i], \quad Mean_{surr} = \frac{1}{M}\sum_{j=1}^{M} d_{surr}[j] \tag{1}$$

$$diff_k = \frac{|Mean_{spec} - \text{Mean}_{surr}|}{Mean_{surr}}, \quad k = 1, 2, \ldots, L, \quad \text{L= number of specularities in an image} \tag{2}$$

$$N_{sm}[k] = \begin{cases} 1 & if \;\; diff_k < threshold \\ 0 & Otherwise \end{cases} \tag{3}$$

$$P_{sm} = \frac{\sum_{k=1}^{L} N_{sm}[k]}{L} \times 100 \tag{4}$$

2 Results

2.1 Quantitative

To compare with the LightDepth (LD) models, in Table 1 we quantitatively evaluate only the sequences used in their study from $Data_{phantom}$ (Cecum 1a, Cecum 2a, Cecum 3a, Sigmoid Colon 3a, Transcending Colon 2a, Transcending Colon 3a, Transcending Colon 4a, Descending Colon 4a). Note that for the Descending Colon, they use half of the sequence, while we use the full sequence. The LD models with UNet and DPT architectures outperform all others (indicated in bold), whereas $SHADeS_{IM,AM,NA}$ still performs best (underlined) among the remaining models. This is expected, as LD was trained on the other phantom sequences and does not require generalization, whereas our model was trained on real data and must generalize when tested on phantom data.

Furthermore, we conduct separate evaluations on the cecum sequences and the remaining anatomical structures, as shown in Tables 2 and 3. Quantitative results are generally worse for the cecum sequences compared to the other anatomical structures (descending, sigmoid, and transcending colon). This is expected, as our real training data did not include any cecum sequences. We also observe that when the sequences are separated in this way, our method is no longer the best in all cases. This suggests that IID may have an advantage in cecum sequences, while MonoViT performs better on the other sequences. However, our method still performs well across all sequences and remains more consistent, which is why it performs better overall.

Methods	Monodepth2	MonoViT	IID	$IID(I_{rem})$	IID_{AM}	$SHADeS_{IM}$	$SHADeS_{IM,AM}$	$SHADeS_{IM,AM,NA}$	LD_{UNet}	LD_{DPT}
$MAE \downarrow$	5.0	5.5	4.9	5.0	5.1	5.6	5.0	4.6	4.4	**3.9**
$MedAE \downarrow$	3.7	3.4	3.7	3.8	3.5	4.3	3.2	3.1	2.9	**2.7**
$RMSE \downarrow$	6.7	8.1	6.9	6.8	7.1	7.7	7.4	6.4	6.3	**5.6**
$RMSE_{log} \downarrow$	0.1922	0.1849	0.2132	0.2122	0.1931	0.2440	0.1801	0.1742	0.1183	**0.1080**
$Abs_{Rel} \downarrow$	0.1596	0.1605	0.1784	0.1782	0.1634	0.2105	0.1510	0.1462	0.0856	**0.0805**
$Sq_{Rel} \downarrow$	1.2208	1.5619	1.7189	1.4810	1.3434	2.4999	1.2900	1.0599	0.0007	**0.0006**
$\delta < 1.25 \uparrow$	0.7595	0.7728	0.7643	0.7495	0.7623	0.7020	0.7873	0.8096	0.9315	**0.9476**
$\delta < 1.25^2 \uparrow$	0.9679	0.9716	0.9332	0.9337	0.9616	0.9113	0.9761	0.9747	0.9934	**0.9965**
$\delta < 1.25^3 \uparrow$	0.9960	0.9982	0.9903	0.9890	0.9929	0.9713	0.9983	0.9974	0.9994	**0.9994**

Table 1: Quantitative depth estimation results (in mm) on selected videos from $Data_{phantom}$, limited to those used in LightDepth (LD) for comparison. The best results are underlined when LD is not included, and displayed in bold when LD is included.

Methods	Monodepth2	MonoViT	IID	$IID(I_{rem})$	IID_{AM}	$SHADeS_{IM}$	$SHADeS_{IM,AM}$	$SHADeS_{IM,AM,NA}$
$MAE \downarrow$	5.6	6.5	**4.7**	4.8	5.6	4.8	5.7	5.5
$MedAE \downarrow$	4.0	3.9	**3.6**	3.7	3.9	3.8	3.8	3.8
$RMSE \downarrow$	7.6	9.4	**6.1**	6.3	7.8	6.3	7.9	7.5
$RMSE_{log} \downarrow$	0.1589	0.1650	0.1493	0.1538	0.1557	0.1568	0.1512	**0.1482**
$Abs_{Rel} \downarrow$	0.1286	0.1408	**0.1211**	0.1249	0.1278	0.1287	0.1295	0.1250
$Sq_{Rel} \downarrow$	1.1969	1.6567	**0.9195**	0.9621	1.1883	1.1054	1.2178	1.1376
$\delta < 1.25 \uparrow$	0.8365	0.8137	**0.8722**	0.8634	0.8497	0.8568	0.8537	0.8606
$\delta < 1.25^2 \uparrow$	0.9916	0.9909	0.9862	0.9850	0.9939	0.9839	0.9943	**0.9968**
$\delta < 1.25^3 \uparrow$	0.9993	**1.0000**	0.9986	0.9978	0.9995	0.9973	0.9997	0.9998

Table 2: Depth estimation quantitative results (in mm) on cecum sequences of $Data_{phantom}$ with best results in bold.

Methods	Monodepth2	MonoViT	IID	$IID(I_{rem})$	IID_{AM}	$SHADeS_{IM}$	$SHADeS_{IM,AM}$	$SHADeS_{IM,AM,NA}$
$MAE \downarrow$	3.6	3.5	4.5	4.4	3.9	4.8	**3.3**	3.4
$MedAE \downarrow$	2.8	**2.3**	2.9	2.9	2.8	3.1	**2.3**	2.4
$RMSE \downarrow$	**5.0**	5.6	7.3	6.9	5.7	7.6	5.1	5.2
$RMSE_{log} \downarrow$	0.1790	**0.1682**	0.2186	0.2131	0.1856	0.2340	0.1693	0.1724
$Abs_{Rel} \downarrow$	0.1473	0.1376	0.1718	0.1681	0.1550	0.1866	**0.1331**	0.1368
$Sq_{Rel} \downarrow$	0.8589	0.9420	1.6062	1.3884	1.0747	1.9894	**0.7790**	0.7985
$\delta < 1.25 \uparrow$	0.7886	**0.8315**	0.7445	0.7508	0.7858	0.7135	0.8268	0.8206
$\delta < 1.25^2 \uparrow$	0.9770	0.9774	0.9386	0.9405	0.9603	0.9282	**0.9775**	0.9757
$\delta < 1.25^3 \uparrow$	0.9981	**0.9982**	0.9872	0.9869	0.9950	0.9810	0.9972	0.9973

Table 3: Depth estimation quantitative results (in mm) on descending, sigmoid, and transcending colon sequences of $Data_{phantom}$ with best results in bold.

2.2 Qualitative

We show additional visual results for estimated depth, albedo, shading, image reconstructions, and specular masks with our method, *SHADeS*, compared to state-of-the-art methods and various ablations. Results for $Data_{real}$ are shown in Figures 1, 2, 3, and 4. Results for $Data_{phantom}$ are shown in Figures 5, 6, 7, and 8.

These extended visual results show that our findings are consistent with those in the main paper, except that our models' albedo colours seem to have limitations with some of the phantom data's unseen textures during training which could be solved with augmentation.

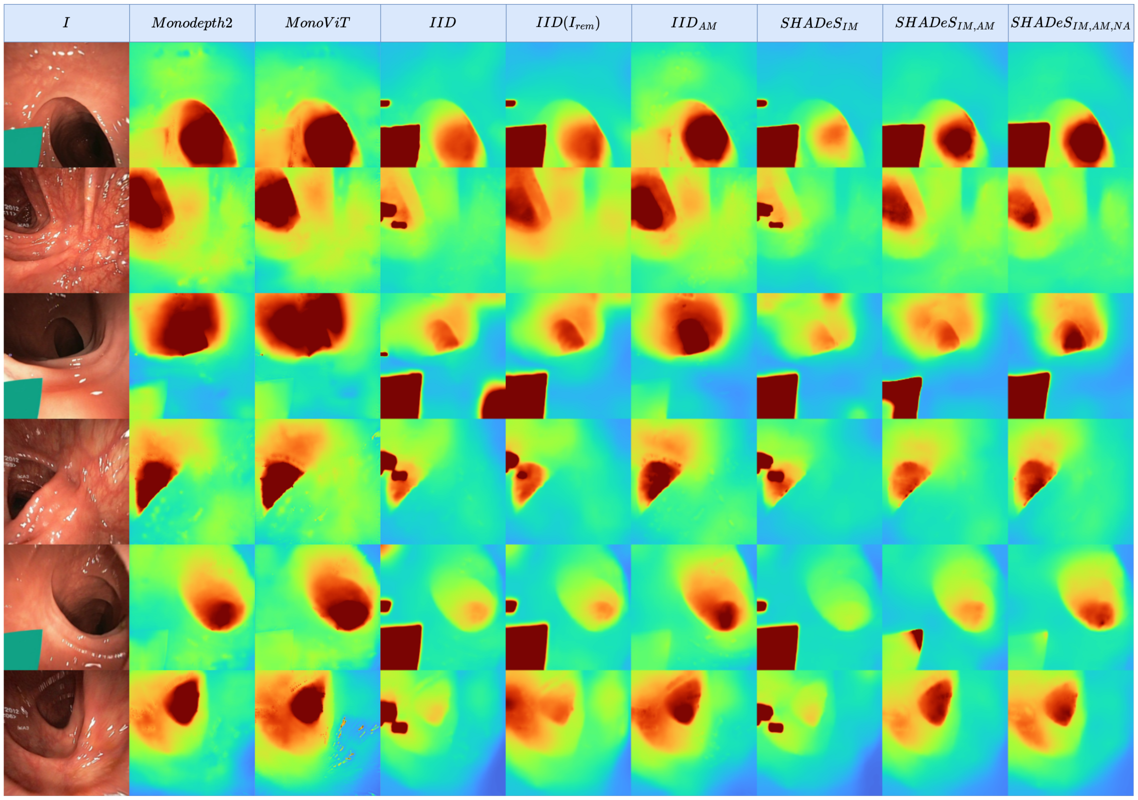

Figure 1: Visual comparison of estimated depth across different methods on $Data_{real}$. For visual clarity, we clip the depth at 0.8.

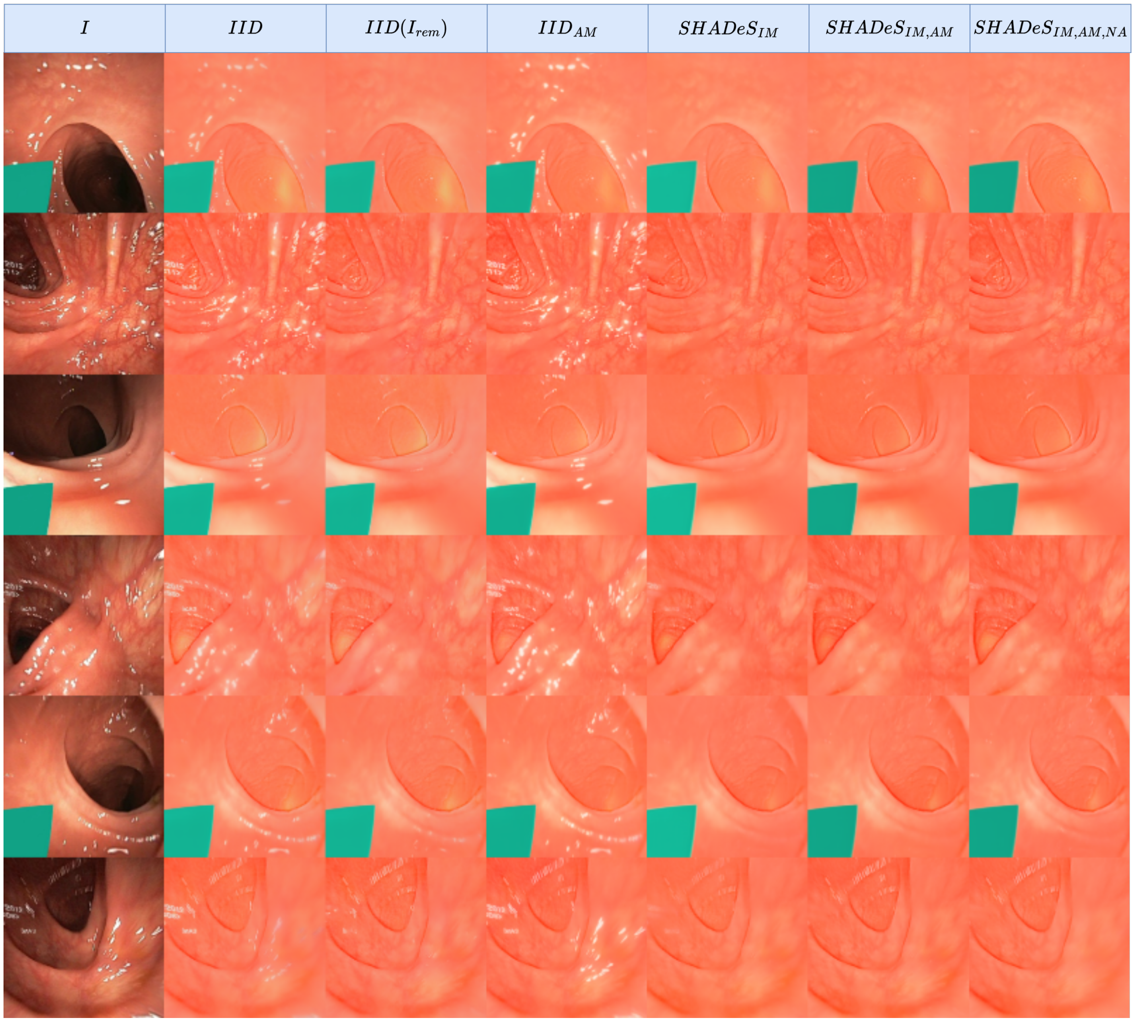

Figure 2: Visual comparison of estimated albedo across different methods on $Data_{real}$.

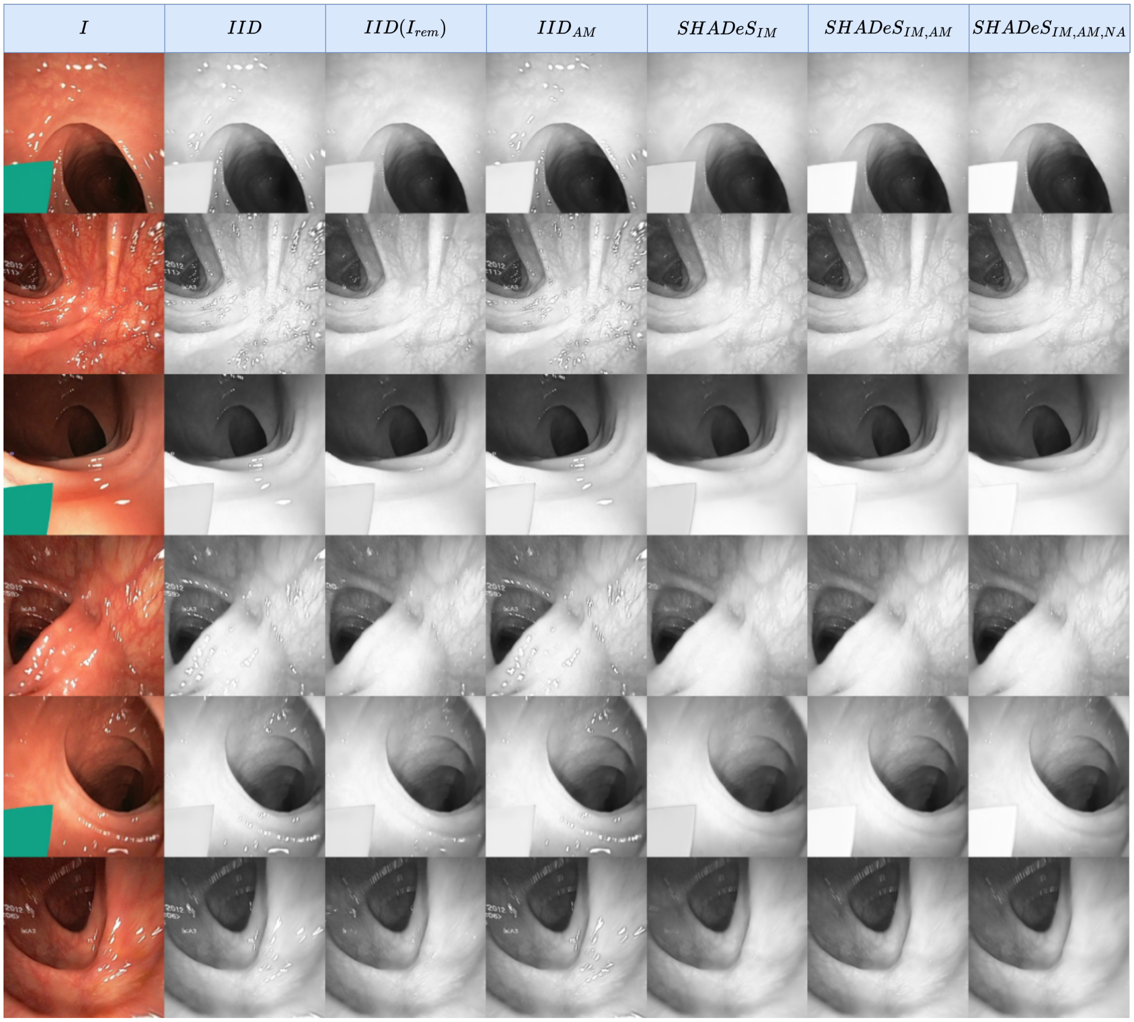

Figure 3: Visual comparison of estimated shading across different methods on $Data_{real}$.

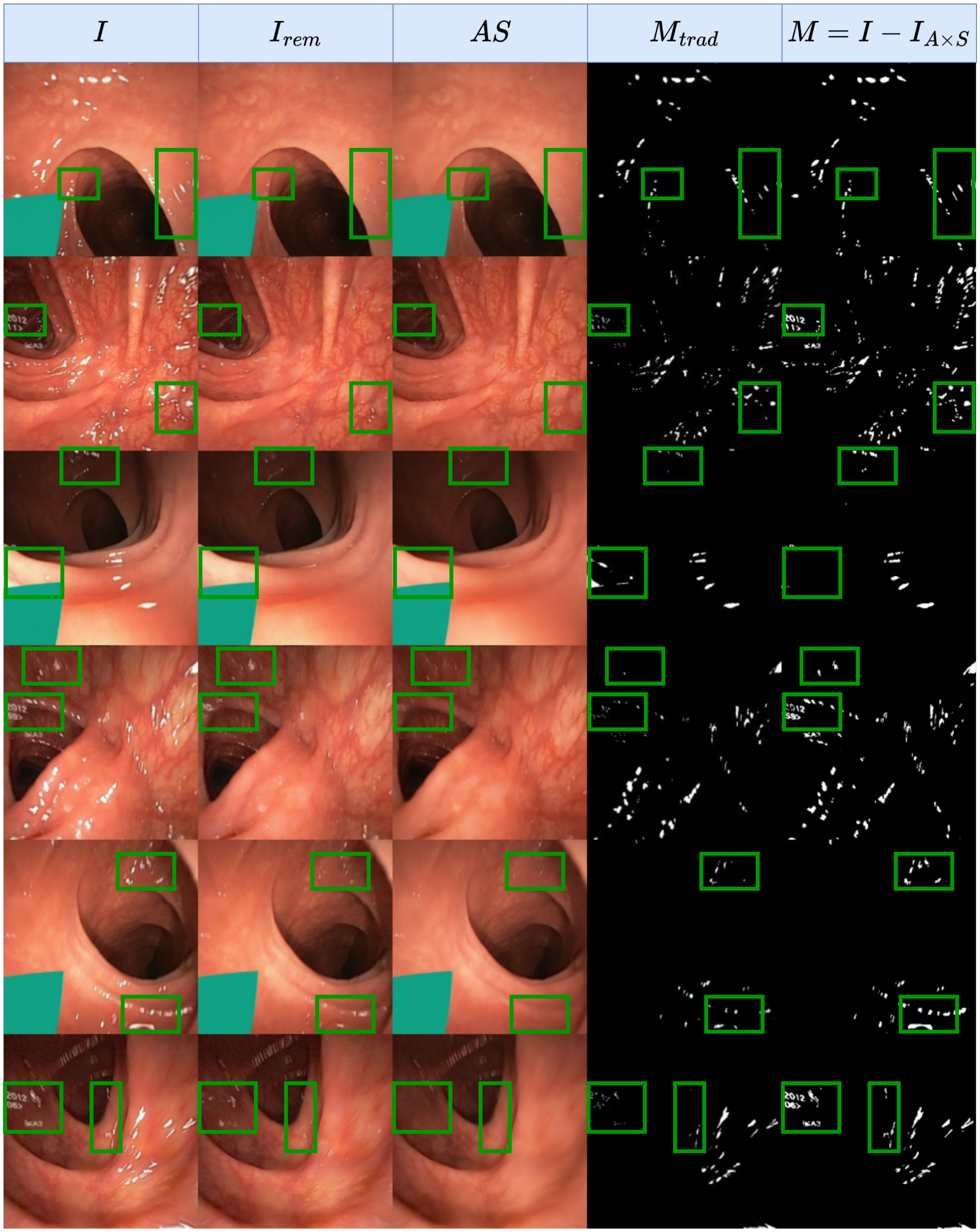

Figure 4: Visualizing $Data_{real}$ images I and the traditional M_{trad} used for inpainting them I_{rem}. We also show the reconstructed images AS and estimated specular masks M from our model $SHADeS$.

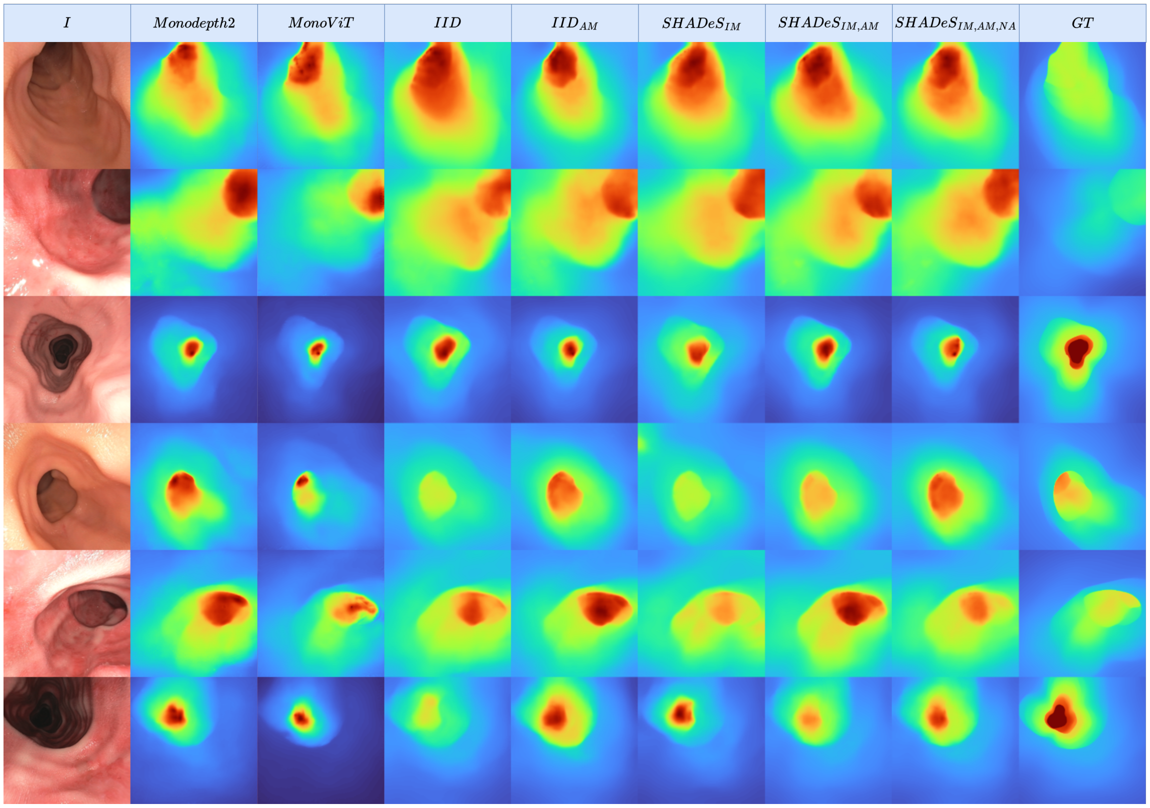

Figure 5: Visual comparison of estimated depth across different methods on $Data_{phantom}$.

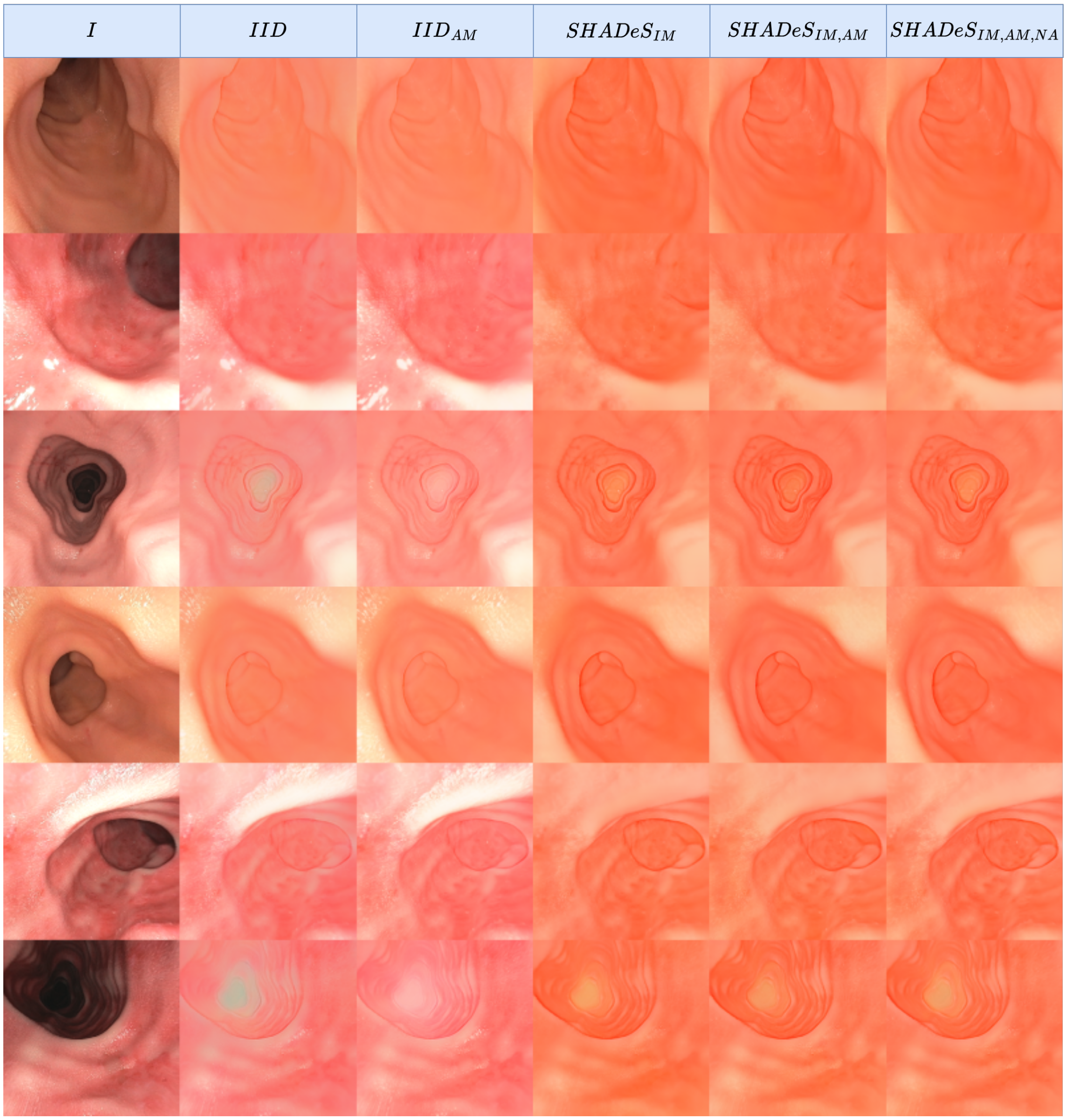

Figure 6: Visual comparison of estimated albedo across different methods on $Data_{phantom}$.

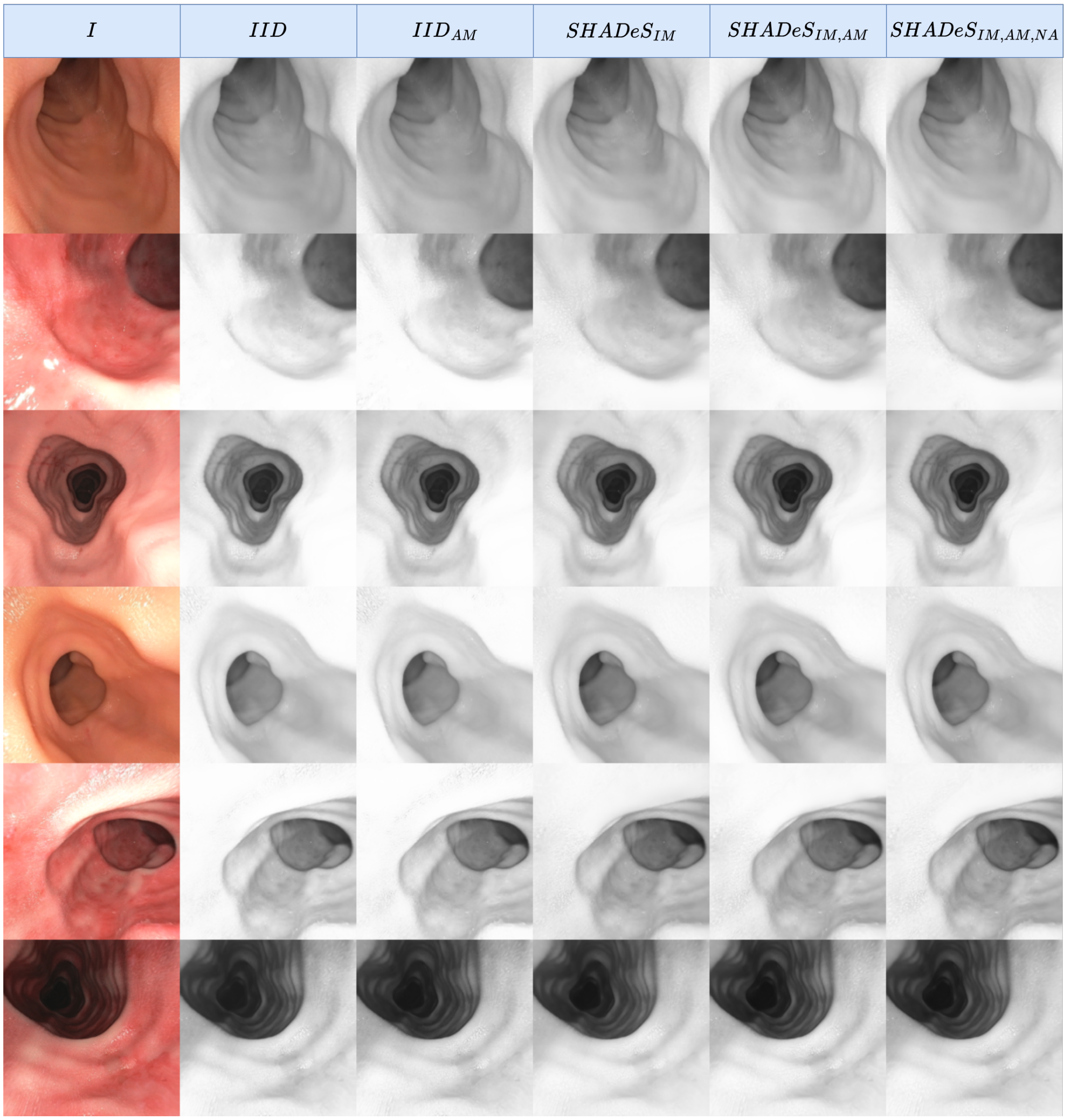

Figure 7: Visual comparison of estimated shading across different methods on $Data_{phantom}$.

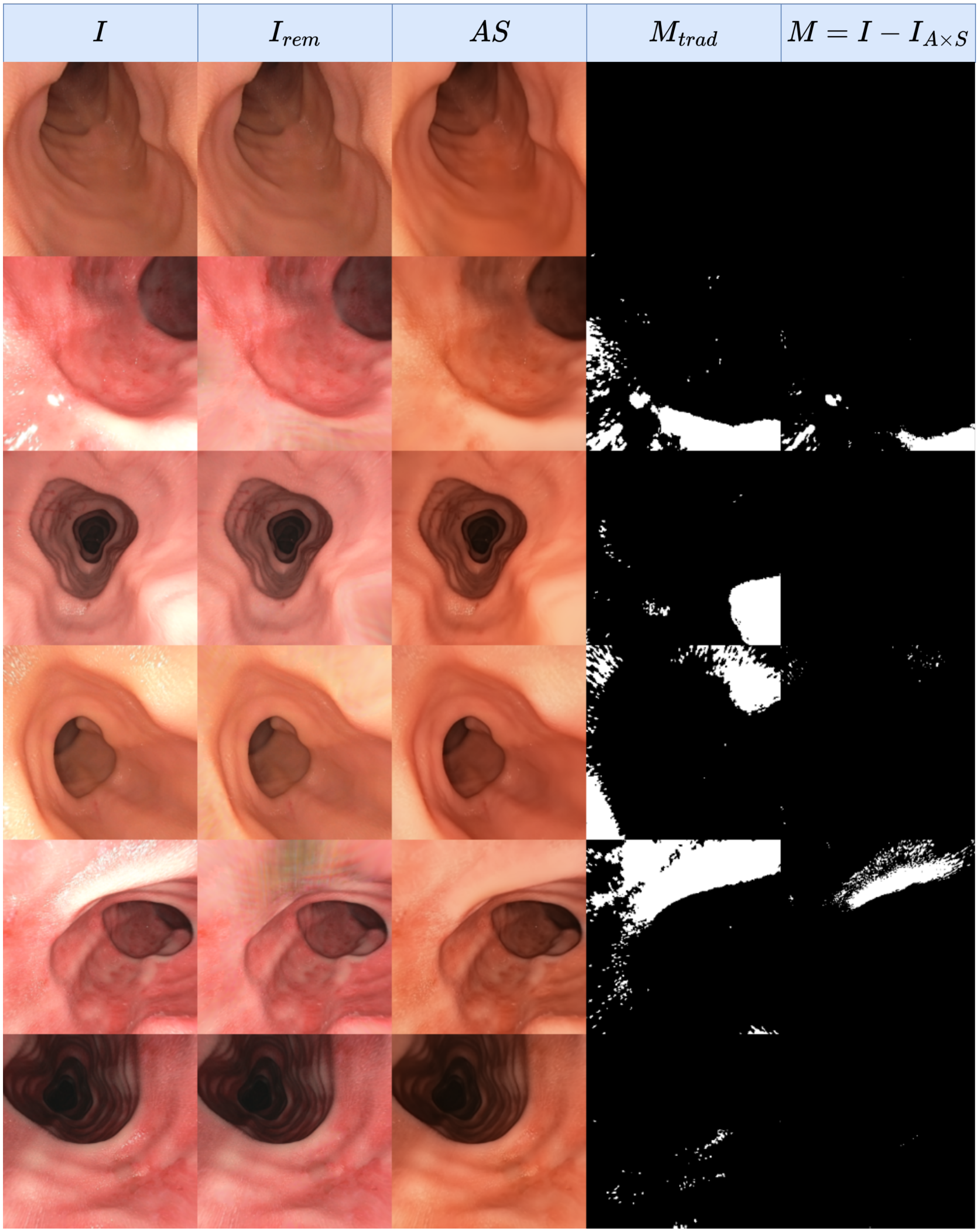

Figure 8: Visualizing $Data_{phantom}$ images I and the traditional M_{trad} used for inpainting them I_{rem}. We also show the reconstructed images AS and estimated specular masks M from our model $SHADeS$.
